# Quality of Life among Next of Kin of Frail Older People in Nursing Homes: An Interview Study after an Educational Intervention concerning Palliative Care

**DOI:** 10.3390/ijerph19052648

**Published:** 2022-02-24

**Authors:** Gerd Ahlström, Helena Rosén, Eva I. Persson

**Affiliations:** Department of Health Sciences, Faculty of Medicine, Lund University, Box 157, SE 221 00 Lund, Sweden; helena.rosen@med.lu.se (H.R.); eva_i.persson@med.lu.se (E.I.P.)

**Keywords:** abduction, next of kin, family members, follow-up study, meaning-focused coping, palliative care, relative, residential housing, qualitative method, quality of life

## Abstract

One cornerstone of palliative care is improving the family’s quality of life (QoL). The principles of palliative care have not been sufficiently applied in nursing homes. The aim of this study was to investigate the experiences of QoL of next of kin of frail older persons in nursing homes after an educational intervention concerning palliative care. This qualitative interview study with 37 next of kin used an abductive design with deductive and inductive content analysis. The deductive analysis confirmed the three themes of QoL from the study before the implementation: (1) orientation to the new life-situation, (2) challenges in the relationship, and (3) the significance of the quality of care in the nursing home. The inductive analysis resulted in the sub-theme “Unspoken palliative care”. Being the next of kin of an older person living in a nursing home can be distressing despite round-the-clock care, so staff need to apply a more explicitly palliative care perspective. Future research needs to evaluate the influence of meaning-focused coping on next of kin’s QoL and integrate this knowledge in psychosocial interventions. Clinical Trial Database for Clinical Research: KUPA project NCT02708498.

## 1. Introduction

Palliative care is seldom initiated before the last day or days of care for older dying persons in nursing homes [1,2], and this can have a negative effect on the next of kin’s quality of life (QoL). Next of kin are an essential part of the older person’s life, and they are involved in the care of the person even when he or she is receiving institutional care [3,4]. Andersson and colleagues [3] found that next of kin were devoted companions of the older person in the last phase of life, participating in the transition towards death. This influences them emotionally, physically, and socially, for which reason the care staff’s understanding of the next of kin is crucial. Enabling next of kin to continue to be involved in the care if it is their wish can increase their well-being and give an improved quality of life for the whole family [5]. Good interaction and communication with staff is a crucial QoL issue for both the next of kin and the older person [6].

The QoL of the next of kin can vary over time, especially in connection with whether the older person’s health is deteriorating or not [7], and it is important that staff have constant open communication with them [1,8]. The time until death after older people’s moving into nursing homes is decreasing [9] and almost one-third die within six weeks after arrival [10]. Behind this lies the policy of “ageing in place”, whereby older persons should be able to live and receive care in their own home for as long as possible [11]. This has led to a situation where moving into a nursing home does not usually occur until the older person is too ill or frail to be able to continue living at home with care from the next of kin and home care services. In addition, the expansion of elderly care has not kept pace with the increased need for care generated by the increasingly older population [9]. A Swedish registry study of 49,172 older people in nursing homes showed that more than half had experienced pain during the last week of life (59%), almost half had experienced wheezing (42%), one-third had experienced anxiety (33%), and slightly more than one-fifth had experienced confusion (22%) [10]. The results indicate the need for improvement of the palliative care to provide relief from different distressing symptoms and better communication about death and dying. This involves prevention of suffering by means of early identification and systematic assessment of physical, psychosocial and spiritual problems [12]. Educational interventions are recommended whereby professionals working in nursing homes can acquire the competence required for meeting the palliative care needs of the older people and their families [8,10,13]. The goal of palliative care is to improve the quality of life of patients and their families facing the problems associated with life-threatening illness. The WHO has called for initiatives to improve palliative care within elderly care, including support for next of kin, and such initiatives need not least to be applied in nursing homes, these being a common setting for end-of-life care [4,12].

Despite the existence of certain interventions to improve palliative care in nursing homes, a systematic review has indicated the necessity for more research to provide a greater understanding of the needs of next of kin [14]. The principles of palliative care have been developed within hospices and specialised palliative care and have not been sufficiently applied in nursing homes [15]. Against this background there was initiated a project called Implementation of Knowledge-Based Palliative Care in Nursing Homes (hereinafter referred to by its Swedish acronym KUPA) [16]. The study protocol [16] describes the implementation strategy, i.e., the educational intervention presented to staff and managers in a series of seminars designed to provide the knowledge and skills deemed necessary to achieve knowledge-based palliative care in nursing homes. The evaluation of the intervention has a non-randomized experimental design with both quantitative and qualitative methods. Baseline and a few follow-up studies are published in respect of staff [17], leaders [18], older persons [19,20,21], and next of kin [22,23,24], and additional follow-ups are in progress. One quantitative follow-up study was based on the World Health Organization QoL questionnaire (WHOQOL-BREF). The results show small improvement of physical health in the intervention group but not in the control group [22]. The current qualitative sub-study within the KUPA project on QoL was designed to give a more comprehensive understanding of the next of kin’s QoL.

Against this background, the aim of the present study was to investigate the experiences of QoL of the next of kin of frail older persons in nursing homes after an educational intervention concerning palliative care. 

## 2. Materials and Methods

This study is a qualitative interview study involving the next of kin of older persons living in nursing homes after the educational intervention within the KUPA project [16]. The semi-structured interviews were performed three months after the educational intervention was completed [23].

### 2.1. Setting

#### 2.1.1. The Care Context of the Nursing Home

The generally accepted definition of a nursing home is that it is a facility that provides 24-h support for people who require assistance with activities of daily living (ADL, i.e., personal and instrumental activities) and have identified healthcare needs. Nursing homes can also play a role in providing palliative hospice care at the end of life [25]. In Sweden, the nursing home is seen as providing frail older persons (>65) with a home of their own. The setting is home-like, with small flats and shared areas. Occupancy is based on a rental agreement signed by the older person or a legally appointed representative of the person. Care and services are provided round-the-clock, and access to a flat is based upon the older person’s needs as assessed by a social worker in the municipality. 

Assistant nurses and care assistants are the main care staff in nursing homes in Sweden, and those provide most of the care to older persons. Assistant nurses (also named enrolled nurses) can have up to three years of vocational education at gymnasium level in elderly care with certain education in palliative care, or have many years of care experience. Care assistants have no training, or only a short training of less than a year, in elderly care. Registered nurses have a broad university education in healthcare to at least Bachelor’s level. They instruct and delegate nursing care and medical tasks to assistant nurses, which can include administering medication and redressing wounds [26,27]. The ratio of RNs to ANs is 1 to 8 and that of ANs to care assistants is 1 to 0.6, i.e., for every AN there are 0.6 care assistants within municipal care including nursing homes [28]. Other professionals with a university degree are registered occupational therapists and registered physiotherapists, who work with rehabilitation and the prescription of assistive devices for the older persons. Physicians from primary health care work as consultants for nursing homes.

#### 2.1.2. Educational Intervention in the KUPA Project

The educational intervention in the KUPA project consisted of five seminars about palliative care at 20 intervention nursing homes and 20 control nursing homes in two counties in southern Sweden [16]. The participants were 8–10 professionals at each nursing home, involving assistant nurses, care assistants, unit managers, registered nurses, occupational therapists, and physiotherapists. Each seminar was held once a month over a six-month period and was carried out at the workplace to facilitate dissemination to the entire work team. The participants received an educational booklet before the start, setting out five themes, and had assignments to be completed between each seminar. The five themes in the booklet were: (1) the palliative approach and dignified care, (2) the role of next of kin and support in palliative care, (3) existence and dying, (4) symptom relief, and (5) collaborative care [16,29]. The booklet and the seminars were based on the existing evidence of good palliative care formulated in two documents issued by the National Board of Health and Welfare [30] and the Regional Co-operative Cancer Centres [31]. These documents are designed to support healthcare providers in developing palliative care and ensuring quality.

### 2.2. Participants

The inclusion criteria were that the person had to be a next of kin of an older person living in a nursing home, regularly visited this older person and was able to understand and speak Swedish. An additional criterion in this study was that the next of kin had participated in the baseline interview nine months earlier, conducted before the start of the educational intervention within the KUPA project [23]. The number of next of kin was dependent on the size of the nursing home, one in the case of smaller nursing homes (<25 older persons) and up to four in the case of the largest nursing homes (>100 older persons); and both urban and rural settings were represented [16]. 

Three of the 40 next of kin who had participated in the baseline interview were drop-outs in this study [23], one due to own ill-health, one due to not having enough energy after the older person’s death and one for no specified reason. The study group of 37 next of kin were between 51 and 85 years old (mean age 64.3 years) and the majority were women, married, a child of the older person, had a college education, were not working and made weekly visits to the nursing home (Table 1). Seven of the 37 interviews were with next of kin of older persons that had died since the baseline interview nine months earlier. 

### 2.3. Interviews and Data Collection

The semi-structured interview guide consisted of three broad questions: (1) How would you describe your quality of life the last two weeks? (2) How do you feel about your situation as next of kin? and (3) To what extent have staff communicated with you about palliative care? The first two of these questions constituted the interview guide used before the intervention [23]. Follow-up questions were asked to deepen the descriptions when the next of kin had more to tell. 

Before the interview, the researchers rang up the next of kin from the baseline interviews [16], giving them information about the study and asking them whether they were willing to continue with their participation. Before the interview began, written information about the study was provided, and written consent from each participant was obtained. Those who agreed to participate chose the time and place for the interview. Most of the next of kin preferred to be interviewed in a conference room at the nursing home or in their own home. 

The interviews lasted between 35 and 90 min (median 42 min). They were conducted by three researchers, all registered nurses with long experience of working in elderly care and palliative care, and were digitally recorded and transcribed verbatim before analysis.

### 2.4. Data Analysis

A qualitative *directed content analysis* in accordance with Hsieh and Shannon [32] was chosen, with both a deductive and an inductive approach. The deductive analysis started from the coding scheme that was generated in the previous baseline interview study within the KUPA project nine months earlier [23]. The analysis concluded with an inductive approach covering data which did not fit in with the coding scheme. Using this abduction with a combination of deductive and inductive analysis is considered more useful in the generation of deeper understanding than using only one of these approaches [33,34]. 

The analysis started with reading the transcripts several times to get an overall understanding of the next of kin’s QoL. Then the meaning units in respect of the next of kin’s quality of life were identified and condensed. A meaning unit is a linguistic unit having similar meaning/content and can consist of a few sentences, a paragraph, or more [35]. The deductive analysis was applied with use of the themes deriving from the interviews with the next of kin conducted before the KUPA educational intervention [23]. The focus was on discovering whether, and if so, in what manner the themes could be identified in the content of the meaning units in these follow-up interviews.

The text not in accordance with the categorisation from the previous study was inductively coded and one new sub-theme was assigned. Two of the authors (HR and EIP) performed the initial analysis and the third author (GA) reviewed the deductive and inductive coding. The final results of the analysis were discussed by all authors until consensus was reached. Selected quotations were used to illustrate the findings. 

## 3. Results

The results are based on the three themes: Orientation to the new life-situation, Challenges in the relationship, and The significance of the quality of care in the nursing home, each with three or four sub-themes (and there is also one new sub-theme which emerged after the intervention) (Table 2). Most of the next of kin described their quality of life in a mainly positive way, even though those of them who were older had different somatic illnesses. They narrated stories of an active social life together with family and friends, and most of them perceived themselves as being in good shape and enjoying travelling. One exception was the time when the older person got worse or was dying. This situation took a lot of energy and time from the next of kin. 

### 3.1. Orientation to the New Life-Situation

The following four sub-themes emerged within the theme *Orientation to the new life-situation*: sense of relief, constantly on one’s mind, feeling alone, and couple’s different life-situations.

#### 3.1.1. Sense of Relief

When a physician or healthcare professional acknowledged that the older person could not live alone at home any longer, the next of kin felt deep gratitude that someone understood how difficult it could be to take care of the older person. Although the older person had been in a nursing home for a time, many of the next of kin, mostly children, had memories of how hard it was when the person lived at home. It was a relief for the next of kin that the older person had attention around the clock and they themselves no longer needed to feel responsible for such things as the shopping and cleaning.

*It’s become easier for me since he came here. Before it was food and stuff, I did everything for him. So it’s become calmer, and they take care of the medicines. I had to do it before, and it’s quite burdensome because it must be right.*

*(daughter, 64 years old)*

The move to the nursing home gave the next of kin more chance to plan their own life, at the same time as they could now appreciate coming to spend time with the older person. Having staff to talk to and share worries with was also a relief. Furthermore the next of kin felt that they played a role at the nursing home by spending time with the other older persons as well.

*It’s such a quiet and nice, gentle time. I like to go there, sit together and talk for a couple of hours, not just with Mum…there are a lot who don’t get visits.*

*(daughter, 68)*

#### 3.1.2. Constantly on One’s Mind

The next of kin could experience burdensome demands when it came to visiting and being with the older person in the nursing home. Usually, it was their own self-imposed demands, and it could be that both staff, friends and relatives tried to encourage them to take a time out from the nursing home. However, it could be just as difficult not to be there because it often meant that they had the older person constantly on their mind, with a bad conscience as a result.

*I’m going off to meet a friend, but I don’t know if I want to, I want to be here. I`ve told the staff that they can call me whenever they want, day and night, if the end is near. I’m terrified that they’ll actually do it when I’m away. It’s difficult, but there’s not much to be done about it.*

*(male spouse, 73)*

Another reason for the next of kin’s spending a lot of time at the nursing home could be that they did not trust the staff to take proper care of the older person. It could also be that they wanted to be at the nursing home to relieve the staff when it came to, for example, feeding the older person, because they felt that the staff had too much to do.

*I go there twice a day, I always go at dinner-time and feed her, and I usually go there and feed her at lunch-time. Because I think the work situation for them [the staff] is that in the evening and at the weekend there are two who are working, and they’ll be feeding four older persons, maybe five sometimes.*

*(son, 53)*

#### 3.1.3. Feeling Alone

Feeling alone could be a question not only of missing the older person but also of missing friends and relatives they normally spent time with. It could also be difficult to summon up the energy to initiate new contacts. Not having siblings or not being in contact with them was also mentioned as a source of loneliness. You can miss having someone who is close to you with whom you can discuss your thoughts and concerns about the older person, miss having someone who can relieve you from having to make all the decisions yourself.

*I’m alone with it because my brother lives in another country. It’s up to me to take Dad to get his eyes seen to, get his hearing aid sorted out, take him to the dentist’s (oh, yes, to the dental hygienist’s as well), take him for eye surgery—it’s all up to me. So it happens that I sometimes feel that it would’ve been nice if I’d had someone I could share this with—but it is as it is.*

*(daughter, 52)*

After living a long life together, the loneliness could be difficult to manage when the spouse had moved to the nursing home. The older person with whom the next of kin had shared many of their thoughts, with whom they had talked about everyday things, was no longer there. It might be that the spouse had dementia or showed no interest due to fatigue or depression.

*It’s the loneliness, and I feel a bit aggressive from time to time. You know, she was the first person I met when I first came here, she was 18 years old.*

*(male spouse, 73)*

#### 3.1.4. Couple’s Different Life-Situations

For spouses, it meant a big change when the partner moved to a nursing home. The one who stayed behind had to take care of practical household tasks that the other partner previously handled and it could be difficult to ask the children for help because they were busy with their own lives, including going to see their father or mother in the nursing home.

*Our kids call me and say, “We’re driving down to see Dad. Are you coming, or … ?” Then I have to cycle there. But nowadays they never come to see me, and it’s natural they go there, but it’s hard to handle. *

*(female spouse, 82)*

There was relief for the next of kin in no longer having to care for their sick spouse and thus getting the opportunity to live their own lives, spend time with friends and relatives. However, the situation also generated frustration and sadness, not only for the older person who needed to move but also for the one who stayed behind. Seeing the spouse living in the nursing home, unable to move or unable to communicate, was painful.

*I’m simply frustrated, as you get when you’d like to talk about something, for example about the children, … You’d like to discuss something but you get no answer. Sometimes it’s almost like I want to shout at him: “But say something, then!”*

*(female spouse, 71)*

### 3.2. Challenges in the Relationship

This theme describes both positive relationships in the sub-theme “enabling good relations”, and complicated relationships in the sub-themes “relationship marked by a guilty conscience”, “encountering the vulnerability”, and “strained relations” (Table 2).

#### 3.2.1. Enabling Good Relations

Spending a lot of time with the older person at the nursing home meant for many of the next of kin that they also had good relationships with the staff, other older persons and other visitors. These persons could become an important part of social life.

*I’d heard about the daughter but never met her before our mothers moved to the nursing home. We’ve a good contact and talk to each other about our concerns, so now I’ve no need for family support. We support and help each other. When she can’t get to the nursing home, I go and see her mother and then I send a text message to tell her [the daughter] about how her mother’s feeling. It’s been great, we also meet in our spare time, with our husbands, it’s really nice.*

*(daughter, 57)*

There could be a heartfelt desire to be there for the older person.

*I’m very happy for my mother, she’s very funny and has always been very funny, so I think it’s fun to come here, even though she’s a little confused sometimes. She recognises me, and it’s great to meet her.*

*(daughter, 55)*

#### 3.2.2. Relationship Marked by a Guilty Conscience

The next of kin could have a bad conscience when the older person imposed demands which were difficult to satisfy. A feeling of inadequacy was often connected with a strong commitment to making the older person’s life as good as possible. Not having the opportunity to offer the older person a certain variety in daily life, such as going out for a walk or going on a car trip, could mean a guilty conscience.

*It’s hard not being able to take him for a car trip like I did the first time here, but the staff said, “Oh, don’t do that anymore, he may not be able to get out of the car.” It’s a pity, because although we never got out of the car we did drive around, and we had coffee with us. He misses those times, when he came out. He just sits there, and he’s actually not that old. *

*(female spouse, 70)*

These feelings could also arise when the next of kin was at home enjoying a meal or socialising with friends.

*I always have a very bad conscience when I’m at home eating a nice meal, I think Dad should have been here, that’s what I think all the time. *

*(daughter, 66)*

#### 3.2.3. Encountering the Vulnerability

It was saddening and worrying to see the older person so vulnerable, to see him or her sad, confused, and sometimes sedated. The difficulty of getting the older person to take medication against anxiety and other discomforts also caused concern. The older person might not understand what the drug was good for. Furthermore there was a fear of seeing the older person with severe dementia, which the next of kin could be worried about inheriting. The older person’s accelerating confusion and anxiety could lead the next of kin to think that he or she had a desire to die. Support from a relative meant a great deal for the next of kin’s well-being, especially during periods when the next of kin was particularly vulnerable, for instance in relation to the older person’s dying.

*My father’s sister was also here when Dad died, and she said “You shouldn’t sit there by yourself”. I felt safe having her with me.*

*(daughter, 66)*

#### 3.2.4. Strained Relations

Strained relations were described in different contexts. Between siblings it could have to do with the fact that one of them took the greatest responsibility and felt there was too little support from the others, or that there had been friction since childhood. It could also be that the older person didn’t want to get involved any longer in what happened at home or in the family.

*The only obstacle is that my husband doesn’t want to move to the nursing home near our home. It was my idea from the beginning that he’d live nearby so that we could see our children, they have their families and they’re busy with their own children. Then they could more easily visit him. Now it’s 70 km to drive, it must be more planned, and we can’t be there so often. We hope that he changes his mind, so we can meet more and talk. We’re a family, even though we live as we live now.*

*(female spouse, 70)*

Furthermore, there could be irritation among the staff because the next of kin did not consider that the older person was properly taken care of, and this could negatively affect the relationship between the next of kin and the older person. The next of kin could find it burdensome to visit the older person when he or she was in a bad mood and blamed the next of kin for one thing or another, or kept expressing a wish to die.

*She’s crying and she’s sad and saying how awful it is and she just wants to fall asleep all the time, and says: “What have I got to live for? If only I could fall asleep for good.”*

*(daughter, 62)*

### 3.3. The Significance of the Quality of Care in the Nursing Home

There was much gratitude and appreciation concerning the care of the older person but there were also those who were less satisfied. The sub-themes were: satisfaction and appreciation, Lack of person-centredness and unspoken palliative care (Table 2). None of the next of kin of a deceased older person used the terms palliative care, symptom relief, end-of-life conversation, or bereavement support/conversation in the interviews.

#### 3.3.1. Satisfaction and Appreciation

Many narratives show how satisfied the next of kin were, both with how they were treated by the staff and with how the older person was taken care of. Life for the next of kin was facilitated by their perception of the staff as being open and welcoming, and when the staff encouraged participation. It meant a lot if the staff did that little extra. The little extra could be the sending of text messages and pictures to say or show that the older person was happy. Or it could be a question of practical things, such as that the older person was especially clean and well-kept and perhaps had had her hair done stylishly.

*He made her so good-looking, she wore her dress, and they’d really worked to make her beautiful. It felt good. They’re very friendly here, preparing her so that she really was part of everything when I came and picked her up.*

*(daughter, 55)*

#### 3.3.2. Lack of Person-Centredness

Even though the next of kin mostly were satisfied with the staff and saw that they worked hard and did the best they could, they were disappointed with the poor continuity of staff. Due to such factors as scheduling and summer holidays there were often many new staff, and the care was not perceived as being person-centred. It could mean that the next of kin had to relate the same things over and over again. Lack of person-centred care could also mean that the next of kin did not feel listened to or that the staff did not show an interest in what was necessary for the older person, which could be frustrating.

*They have a new doctor here, and we’re not really on the same wavelength. Still, I’m my mother’s voice.*

*(daughter, 61)*

A reduced labour force made it difficult for the staff to do that little extra. Moreover, the staff did not always show an interest in the older person’s past life, interests and spirituality. It could be, for instance, that the older person wanted to participate in a religious service but got no help getting there in their wheelchair.

#### 3.3.3. Unspoken Palliative Care

This sub-theme emerged from the inductive analysis. The next of kin spoke about the older person’s poor health even before the move to the nursing home, saying that they had wanted the older person to receive a place earlier. The next of kin were more or less aware that the older person was going to die at the nursing home, and this awareness increased as the older person’s health worsened. They saw the worsening as natural and expected. The next of kin trusted the staff, who did their best and were kind to the older person.

*I was always welcome when I went there and they’re very good staff. The last time for me with my dad, … I can’t think of any better way to die than the way he did.*

*(daughter, 66)*

The next of kin emphasised the importance of the older person’s not being alone at the time of death. They were there as much as they could at the end, but not all of them did succeed in coming in time for the older person’s death. When they were not present themselves, they appreciated that there was someone they had confidence in among the staff. Some of them had a strong memory, for instance, of the moment when the staff said that the older person fell asleep, calmly and quietly, and they felt gratitude for the peaceful death. The moment of death could come too quickly and suddenly, even though it was expected that it would happen soon.

*I’d fallen asleep in the room where my mother was lying. Then I woke up, ten to three in the morning, and saw that Mum was taking her last breaths, she was breathing irregularly. Nobody woke me up, but something told me that now was the time to wake up. I sat up and held my mum’s hand, and she died after a quarter of an hour. Those special breaths… I called my brother and said “You have to hurry, come here”, and I cried because I didn’t want to be alone. But he didn’t understand, so I was alone when it happened.*

*(daughter, 51)*

The next of kin’s experiences revealed a frustration over not receiving enough information about the process of dying. The physician’s telling them that death was approaching came at a late stage but was meaningful and indeed essential for them.

*One nurse called my brother, and he called us because they thought she was very, very ill. So we went all there, but then it was not the end … Then she [the physician] said, “Now there are only a few days left” and she said quietly and calmly that now we needed to make the most of those days.*

*(daughter, 65)*

## 4. Discussion

### 4.1. Results Discussion

The findings of this study revealed that the next of kin’s QoL included both positive and negative emotions, sometimes co-occurring in certain stressful situations. It was a relief for the next of kin when the older person had attention in the nursing home round the clock and it made it easier for them to have their own active social life. The next of kin’s separation from the loved-one called for an orientation to the new life-situation, and generated challenges in the relationship that also involved other family members, and the significance of the quality of care in the nursing home became an essential issue for their well-being. Despite their own ailments the next of kin were relatively satisfied with their QoL. A rather high age in the study group can explain their concerns about their physical health. Two-thirds of the next of kin were 60 or older, even though most of them were children of the older persons living in the nursing home. In addition, the results show that the next of kin did not talk about palliative care in the interviews and indeed did not understand its meaning before the older person’s death.

**Figure 1:**
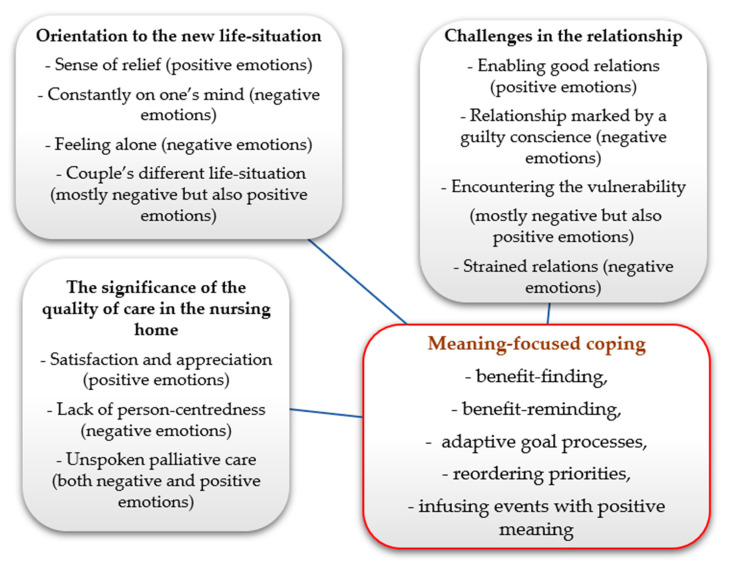
The results regarding stress and emotions from the perspective of meaning-focused coping.

The next of kin’s QoL in this study can be understood from the theoretical perspective of stress and coping [36]. Aspects of stress or negative emotions are described in all three themes, as illustrated in Figure 1. The experience of stress is a dynamic cognitive process that starts when a person identifies a situation or event as a hurt (loss), threat or challenge. The well-known ways of handling stress are problem-focused and emotion-focused coping [36,37]. Problem-focused coping is directed towards the source of the stress and consists mostly of actions, whilst emotion-focused coping is directed towards regulating emotions occasioned by the stress [36,37,38]. In this study, the next of kin described the time before the move, when they were responsible for the care and all practical work with the household. This was a period with a lot of demands involving problem-focused coping that lasted up to the point where the older person’s health had deteriorated so much that a move to a nursing home became the inevitable choice, the only feasible form of problem-focused coping that was left. Previous research has shown that during the period leading up to a nursing home placement the older person is often in need of comprehensive care and assistance in their ordinary home [9,39]. The next of kin’s situation has been described in terms of double feelings: there is relief at escaping from the strenuous situation as caregiver, and there is bad conscience about being the driving force behind the older person’s move [23,40,41].

The situation for partners and children of parents in this study is that a longstanding close relationship with an older person has been broken up after the move to a nursing home, giving rise to a stressful situation for the whole family. They can be seen as engaging in emotion-focused coping. However, in this study we assess these feelings as meaning-focused coping, which is a further development from the theory of stress and coping set forth by Susan Folkman [37]. This development derives from research on next of kin through the observation that positive emotions and negative emotions co-occur during the intensely stressful experience of caring for a severely ill loved one [37]. Our interpretation of the results of this study on the basis of the concept of meaning-focused coping is shown in Figure 1.

According to previous research, meaning-focused coping generates positive emotions in which the person draws on his or her beliefs (e.g., religious, spiritual, or concerning justice), life values and existential goals to motivate and sustain well-being during a difficult time [37,42]. The assigning of a positive meaning to an event can result in a strengthening of social and personal resources, contributing to the person’s adjustment [43]. However, more studies are needed to broaden the scope of this field of inquiry with particular reference to the next of kin of older persons in nursing homes.

The deductive analysis revealed that the themes from the study carried out before the intervention [23] were suitable for application to the interview data in the present study. However, one new sub-theme emerged from the inductive analysis: unspoken palliative care. This was related to the older person’s death that had occurred during the time between the two interviews. The next of kin’s awareness that the older person was going to die increased when the older person’s health worsened. Whittaker and colleagues [13] found that more than half of the staff in nursing homes (59% of 119) were not familiar with the principles of palliative care. In paediatric palliative care the unspoken aspect was a question of incomplete communication stemming from the parental wish to withhold information from the child [44]. This misguided protection resulted from a fear of hurting the sick child and not causing them worry [45]. It is plausible that there may exist similar misguided protection on the part of the next of kin of frail elderly people in nursing homes. Dahlborg, Lyckhage and Lindahl [46] interviewed six next of kin of terminally ill older persons living at home. They mean that the unspoken aspect requires staff to take responsibility for identifying and responding to the situation. As an increasing number of people end their lives in nursing homes, palliative care has increasing relevance. We recommend a palliative care educational training programme, taking into account the QoL of next of kin. Improved communication about psychosocial aspects between staff and next of kin should prove not only their own mutual understanding but also that of the next of kin and the older person. We focus in the following text on comparisons of the results concerning QoL of next of kin between this study and the one undertaken before the educational intervention. The two studies can be seen as forming a whole when it comes to understanding QoL among the next of kin of frail older persons. Previous research [23,41] supports our finding that the next of kin feel relief after the older person has moved to a nursing home. Regarding “encountering the vulnerability”, this was evident both before [23] and in this study three months after the educational intervention, which can be attributed to the fact that the next of kin face the older person’s emotional experiences involving a loss of interest in life and sometimes a desire to die. However, in the first study [23] the next of kin expressed more frustration and a greater sense of their own inadequacy, while in the present study they expressed more emotional strain, sadness and worry. This may imply that the next of kin are going through different stages of transition, the first being more a question of processing and the second being more a question of incorporation [37,47]. There is a similarity between this study and the earlier one [23] in that “relationship marked by a guilty conscience” could appear on certain occasions, such as when the next of kin was enjoying good food or socialising with friends without the older person. In the earlier study, however, guilty conscience also appeared without real cause when there was an inability to cope as the older person’s condition worsened or the older person became more demented, was unable to speak, lived in the past or behaved rudely. In the present study, there more clearly emerges a concern about not having the opportunity to offer the older person a certain variety in daily life. In both studies, the “enabling good relations” sub-theme had to do with safeguarding the older person. In this study, it also had to do with establishing good relationships with other older persons and their next of kin. Strained relations occurred in both studies, often described as having lingered a long time and even from childhood between siblings or between children and parents. There was revealed an irritation at the staff’s not taking care of the older person properly. The findings of both studies revealed satisfaction and appreciation when the relationship to the older person and the staff was good, and in the present study the appreciation of other visitors was a further positive experience. Lack of person-centredness in both studies covered disappointment that the older people didn’t receive the person-centredness they deserved in the autumn of their lives. The expressed disappointment concerned poor continuity of staff, often due to such factors as scheduling and summer holidays. The understanding of the QoL of the next of kin was deepened by comparing the results of the present study with the results before the intervention, which both confirms certain of their subjective experiences and shows that there have been changes in others. The review study by Andershed [48] revealed that next of kin of older persons in end-of-life (EoL) care are in a burdensome situation involving increased vulnerability. They are quite often unfamiliar with the issues they are facing, insecure and uncomfortable about what to say, what to do, how to handle such a situation [49]. The results of the present study confirmed this in that the term palliative care and such related terms as symptom relief, end-of-life conversation and bereavement support were not used by the next of kin, not even when the interviewer used them. The next of kin described frustration over the lack of information about the process of dying and over the physician’s not telling them until a late stage that the older person was approaching death. Traditionally, the principles of palliative care have been developed within hospices and specialised palliative care for hospitalised patients with advanced-stage cancer; and older persons with multi-morbidity in nursing homes and their next of kin have so far received less palliative care [12]. For this reason, the KUPA project was designed to develop the palliative care for the elderly by improving the knowledge base in respect of everyday care for staff and managers [16].

Most of the staff who participated in this study had limited training in palliative care, this could be a reason why the intervention is not clearly reflected in the QoL of the next of kin. A recently published study within the KUPA project shows that the assistant nurses and care assistants had large expectations with regard to gaining increased knowledge in palliative care before the start of the educational intervention [17]. According to Froggatt [50] education and training are not enough to bring about sufficient changes in practice in nursing homes. Cultural change is needed, as described in various studies [50,51,52,53]. Here good leadership can be one of the key elements in developing the care.

In the KUPA project, the managers of the nursing homes participated in the seminars aimed at imparting knowledge and skills to increase staff confidence and competence in providing palliative care [18].

### 4.2. Methodological Considerations

This study has both limitations and strengths. The fact that the study group comprised next of kin who almost all visited the older person at least weekly and thus maintained an emotional relationship with him or her strengthens the trustworthiness of the results together with the results from the study before the educational intervention [23]. However, one weakness of the study is that the study group consisted mostly of women. One possible explanation is that the staff were almost all women (95.5%), as were the contact persons for the project: they were perhaps more prone to ask next of kin of the same gender about participation in the study. Gender has been found to influence the interaction in qualitative interviews, where participants of the same gender seek similarities and differences based on their similar socio-cultural expectations [54]. However, other studies have found the opposite, where men are equally or even more comfortable speaking to a woman compared with speaking to a man [54,55]. Our own clinical experience indicates that daughters are the most frequent visitors of older persons in a nursing home, which probably explains the overrepresentation of women in the study group. The literature is unequivocal regarding women as the most common caregivers [56,57]. This means that the results of the study cannot automatically be applied to men, which is a weakness concerning transferability that has to be taken into account. The three researchers who performed the interviews had comprehensive competence in talking with next of kin, based on their clinical experience as nurses in geriatric and palliative care. In order to ensure the dependability of the results there were weekly meetings with the interviewers during the whole data collection, led by the project manager. These gave plenty of opportunities for calibrating the interview guide and adjusting the way of conducting the semi-structured interviews.

The abductive design, mostly neglected in nursing research, reflects the process of generating hypotheses, theories, or explanations [33,34]. Abduction represents a type of combination or synthesis of deduction and induction. In this study, the results were furthermore framed within the theoretical perspective of meaning-focused coping. This framework represents a promising theoretical perspective needing to be tested in support programmes for next of kin.

Similar studies in the literature make use only of interviews after the intervention. However, prolonged data collection with repeated interviews ensures the dependability of the results [58,59]. In the case of the current project the previous study involved analysis of the interviews before the intervention [23] whilst the present study involved a two-stage analysis: first deductive, directed towards the themes from the previous study, then inductive, directed towards text not covered by these themes. The chosen method was directed content analysis [32], which strengthens the dependability and credibility of the conclusions regarding possible changes in QoL. Hsieh and Shannon [32] assert that the goal of directed content analysis is to validate or to expand a theoretical framework or theory in respect of a phenomenon that would benefit from further description. In the present study, the themes and sub-themes were those of the pre-intervention study [23], except that one new sub-theme emerged in the inductive phase of the analysis. This indicates a high dependability and credibility of the results.

Investigator triangulation was applied to ensure credibility in the analysis [59]. Two independent researchers (second and third authors) performed the preliminary analysis from the coding manual as well as the inductive analysis at the end. In the next step, the first author read the interviews, reviewed the categorisation and suggested alternative interpretations of the text. Thereafter, the results were discussed at several meetings before consensus was reached about their final form. This was designed to increase their trustworthiness.

## 5. Conclusions

The findings of this study revealed that the QoL among next of kin included both positive and negative emotions, sometimes existing together in the same stressful situation. Being the next of kin of an older person living in a nursing home is significantly distressing despite the care around the clock. The new sub-theme “unspoken palliative care” had to do with the fact that the next of kin did not refer to palliative care in the interviews, which indicates that they had limited awareness that the older person was going to die. Nursing-home staff need to give increased attention to applying a palliative care perspective and exploring the next of kin’s resources within the framework of stress and meaning-focused coping. Future research needs to further investigate the influence of meaning-focused coping on the next of kin’s QoL, and the resulting information should be used to initiate psychosocial interventions within palliative care to improve this QoL.

## Figures and Tables

**Table 1:** Characteristics of the participating next of kin (*n* = 36, data missing on one next of kin).

	N (%)		N (%)
Age, years		Educational level	
50–59	10 (27.8)	Compulsory school	7 (19.4)
60–69	17 (47.2)	Secondary school	10 (27.8)
70–79	7 (19.4)	Trade school	3 (8.3)
80–89	2 (5.6)	University/college	16 (44.5)
Gender		Work status	
Men	7 (19.4)	Full-time	11 (30.6)
Women	29 (80.6)	Part-time	7 (19.4)
Marital status		Not working	18 (50.0)
Married/living together	26 (72.2)	The frequency of visits	
Unmarried/divorced	7 (19.4)	to the old person	
Widower/widow	3 (8.3)	Every day	4 (11.1)
Relation to the older person		Weekly	30 (83.3)
Husband/wife	7 (19.4)	≤Monthly	2 (5.6)
Daughter/son	27 (75.0)		
Sibling	1 (2.8)		
Other	1 (2.8)		

**Table 2:** Themes and sub-themes of quality of life among the next of kin of older persons living in nursing homes (*n* = 37).

Themes	Sub-Themes
***Deductive analysis*** [23]	
Orientation to the new life situation	Sense of relief
	Constantly on one’s mind
	Feeling alone Couple’s different life-situations
Challenges in the relationship	Enabling good relations
	Relationship marked by a guilty conscience
	Encountering the vulnerability
	Strained relations
	Satisfaction and appreciation
The significance of the quality of care in the nursing home	Lack of person-centredness
***Inductive analysis***, the new sub-theme	Unspoken palliative care

## Data Availability

The datasets used and analysed during the study are available from the project leader (G.A.) upon written request and in accordance with ethical approval.
